# Unveiling the Impact: A Scoping Review of the COVID-19 Pandemic’s Effects on Racialized Populations in Canada

**DOI:** 10.3390/ijerph22071054

**Published:** 2025-06-30

**Authors:** Menna Komeiha, Iryna Artyukh, Oluwasegun J. Ogundele, Q. Jane Zhao, Notisha Massaquoi, Sharon Straus, Fahad Razak, Benita Hosseini, Navindra Persaud, Sharmistha Mishra, Azza Eissa, Mathieu Isabel, Andrew D. Pinto

**Affiliations:** 1 Upstream Lab, MAP, Li Ka Shing Knowledge Institute, Unity Health Toronto, Toronto, ON M5B 1T8, Canada; menna.komeiha@unityhealth.to (M.K.); iryna.artyukh@unityhealth.to (I.A.); jkoluwasegun@outlook.com (O.J.O.); benita.hosseini@unityhealth.to (B.H.); azza.eissa@mail.utoronto.ca (A.E.); 2 Institute for Health Policy, Management and Evaluation, Dalla Lana School of Public Health, University of Toronto, Toronto, ON M5T 3M6, Canada; jane.zhao@uhn.ca (Q.J.Z.); sharon.straus@unityhealth.to (S.S.); fahad.razak@unityhealth.to (F.R.); 3 Department of Health and Society, University of Toronto Scarborough, Scarborough, ON M1C 1A4, Canada; notisha.massaquoi@utoronto.ca; 4 Department of Family and Community Medicine, St. Michael’s Hospital, Toronto, ON M5B 1W8, Canada; nav.persaud@unityhealth.to; 5 Keenan Research Centre of the Li Ka Shing Knowledge Institute at St. Michael’s Hospital, Toronto, ON M5B 1W8, Canada; 6 Knowledge Translation Program, Li Ka Shing Knowledge Institute at St. Michael’s Hospital, Toronto, ON M5B 1W8, Canada; 7 Geriatric Medicine, Department of Medicine, University of Toronto, Toronto, ON M5S 3H2, Canada; 8 Department of Family and Community Medicine, Faculty of Medicine, University of Toronto, Toronto, ON M5G 1V7, Canada; 9 Dalla Lana School of Public Health, University of Toronto, Toronto, ON M5T 3M7, Canada; sharmistha.mishra@utoronto.ca (S.M.); mathieu.isab@gmail.com (M.I.); 10 Department of Medicine, University of Toronto, Toronto, ON M5S 1A1, Canada; 11 Institute for Clinical Evaluative Sciences, Toronto, ON M4N 3M5, Canada; 12 Family Medicine Department, Université de Montréal, Montréal, QC H3T 1J4, Canada

**Keywords:** COVID-19, disadvantaged populations, racialized, Indigenous, equity, discrimination

## Abstract

Objectives: The objective of this study was to examine the impact of the COVID-19 pandemic on racialized communities and individuals in Canada. Methods: This review followed the Joanna Briggs Institute (JBI) methodology and the PRISMA-ScR guidance on reporting scoping reviews. Ovid MEDLINE ALL, Embase Classic + Embase, CINAHL (Ebsco platform), PsycINFO, and Cochrane were searched for documents that were published after March 2020 and that reported on the social and economic impacts and health outcomes of the COVID-19 pandemic on generally healthy racialized populations that reside in Canada. Synthesis: A total of 39 documents were included in this review. Our results show racialized communities faced greater social, economic, and health impacts from the pandemic. These impacts were manifested in the form of high COVID-19 morbidity and mortality rates, increased discrimination, worsening mental health, difficulty in accessing healthcare, and challenges related to accessing food and basic necessities. Conclusion: Canadian racialized groups have been inequitably affected by the COVID-19 pandemic due to pre-existing inequalities and emerging discrimination. Responsive policy action and robust pandemic preparedness efforts are indispensable in adopting a proactive stance to prevent racialized populations from bearing a disproportionate burden of negative health crises in the future. This necessitates addressing pre-existing disparities and targeting social and economic vulnerability areas. By doing so, we can mitigate the reported social, economic, and health impacts experienced by racialized groups, including challenges related to accessing basic necessities, deteriorating mental health, and barriers to healthcare access.

## 1. Introduction

Early in the pandemic, COVID-19 was hailed a “great equalizer” by some journalists and politicians [1]. However, evidence from the literature showed that racialized individuals were disproportionately impacted by past pandemics [2,3]. During a pandemic, racialized communities often experience higher rates of poverty, unemployment, educational disparities, and encounter barriers in accessing healthcare [4,5,6]. Disparities in wealth and resources due to discriminatory practices of educational, government, and healthcare systems—known as structural inequities—usually worsen during a public health emergency [7,8].

Structural inequities, rooted in systemic racism, not only amplify the disproportionate impact of pandemics on racialized communities but also perpetuate health disparities through interconnected social determinants such as housing, education, and employment. Racial discrimination manifests in exclusionary housing practices that perpetuate segregation and economic disparities [9,10]. Housing is a social determinant of health (SDoH) that significantly influences obtainable opportunities such as education and subsequent employability prospects [11,12]. In Canada, school zoning and funding are based on property taxes [13,14], leading to disparities in housing values and educational resources that affect employment opportunities and income levels, further deepening health inequities.

High-poverty schools, often serving racialized students, faced systemic barriers like inadequate funding and resources, hindering access to technology and quality remote instruction [15,16]. The shift to virtual interactions exposed racialized populations to heightened online discrimination [17]. The pandemic also exacerbated job market challenges, and financial instability [18,19].

In this scoping review, “racialized identities” are understood as groups of people socially and politically constructed as “racially” distinct [20]. These identities are historically and contextually specific, characterized by their malleability and situational nature [20]. In the Canadian context, and for the purposes of this study, “racialized” refers to individuals who do not identify as White.

Recognizing the links between systemic racism, SDoH like income and employment, and the pandemic’s disproportionate impact on racialized communities, this scoping review seeks to enhance understanding of how the pandemic has affected the health, social, and economic well-being of racialized individuals in Canada.

### Objectives

This scoping review aims to examine the impact of the COVID-19 pandemic on racialized communities and individuals in Canada by addressing the research question: How did COVID-19 impact racialized communities and individuals across Canada?

## 2. Methods

We conducted a scoping review in accordance with the Joanna Briggs Institute methodology for scoping reviews [21] and PRISMA-ScR guidance [22] (Supplementary File S1—PRISMA–ScR checklist) to assess the impact of COVID-19 on racialized groups in Canada, including economic, health and social consequences. A scoping review was deemed appropriate due to the expected heterogeneity of the primary data.

The protocol for this scoping review was registered and published on the Open Science Framework [23].

### 2.1. Eligibility Criteria

This review focused on documents whose focus was on Canada involving generally healthy adults who identify as racialized. Eligible documents assessed the COVID-19 pandemic’s impact on these populations and were published in English after March 2020, following the World Health Organization’s pandemic declaration.

To focus specifically on the broader population’s experiences during the COVID-19 pandemic, we excluded documents involving healthcare workers and individuals with pre-existing comorbidities, as these factors could confound the observed impacts due to heightened exposure risks or underlying health conditions. Additionally, we excluded documents on animals, opinion pieces, commentaries, panel discussions, conference abstracts, news articles, case reports, and those focused on pediatric populations. Systematic reviews were included when relevant. The literature search was limited to documents published up to December 2023.

### 2.2. Information Sources

An information specialist (KE) developed and tested the search strategies in collaboration with the review team. The literature review was conducted in two phases.

In phase one, a systematic search was conducted across the following databases: Ovid MEDLINE ALL, Embase Classic + Embase, CINAHL (Ebsco platform), PsycINFO, and Cochrane. The search strategies combined controlled vocabulary (e.g., coronavirus infections) and keywords (e.g., racialized), with adjustments made to vocabulary and syntax across the databases (Appendix A). Documents nominated by team members that met the inclusion criteria were considered if they had not been previously identified.

In phase two, a search of grey literature sources was conducted using pre-defined keywords to supplement the initial results. Grey literature repositories searched included Health Canada, Health Quality Ontario, the National Collaborating Centre for Public Health, and the Canadian Institute for Health Information. To enhance the search, the same set of pre-defined keywords was used in a Google search, screening the first 100 results for each keyword (Appendix B). The full electronic search strategy can be found in Appendix A.

### 2.3. Search Strategy

We utilized the Population, Phenomena of Interest, Context, Outcome, and Time frame (PICOT) framework to create a structured search strategy. The components were defined as follows: Population of interest (P): racialized populations; Phenomena of interest (I): the health, social, and economic impacts of COVID-19; Context (C): the impacts of COVID-19 on racialized populations in Canada; Outcome (O): impact assessment focusing on the SDoH; Time frame (T): March 2020 to December 2023. This period captures the onset of the pandemic, the subsequent waves, and recovery efforts, allowing for an analysis of both immediate and longer-term effects.

### 2.4. Selection of Sources of Evidence and Data Extraction

A two-stage screening process was conducted using Covidence. First, titles and abstracts were screened by two independent reviewers (MK, IA, HL). Full-text screening followed for eligible documents, with conflicts resolved by a third reviewer (OO). Quality appraisal was not conducted, as this review aimed to capture the breadth of COVID-19’s impact on racialized communities. Given the limited number of documents systematically collecting race-based data, all available sources were included regardless of quality to ensure a diverse range of perspectives.

The reviewers participated in practice exercises and then independently extracted study details. Data extraction was conducted by two reviewers (MK, IA) using a pretested data extraction template (Appendix C). A standardized coding protocol was followed to collect information such as the author, study title, year, setting, design, methodology, study population, and reported impacts.

### 2.5. Synthesis of Results

We employed Popay et al.’s [24] narrative synthesis approach to integrate the collected data, given the heterogeneity of the included articles. Our review examined a wide range of impacts, extending beyond morbidity and mortality to encompass various socioeconomic effects.

### 2.6. Synthesis

**Figure 1:**
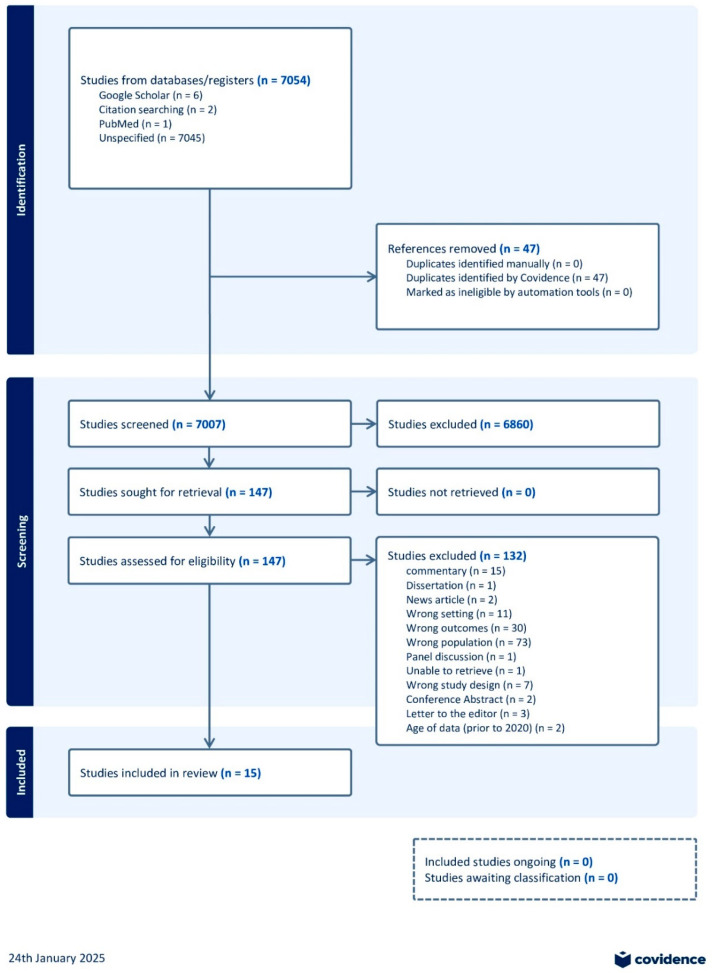
PRISMA flowchart of peer-reviewed articles.

The search identified 7054 citations. After removing 47 duplicates and conducting title and abstract screening, 147 documents were selected for full-text review. Fifteen documents examining the impact of COVID-19 on racial populations were identified, along with twenty-four reports from grey literature sources (Figure 1. PRISMA flowchart of peer-reviewed articles).

The included documents varied in research design. Four documents adopted mixed-method approaches, three used qualitative methodologies, one was a systematic review, and seven were quantitative investigations (Table 1 and Table 2). Additionally, 24 grey literature articles were included (Table 3). These documents and articles had diverse objectives, including reporting on experiences of racism, understanding disparities in healthcare access, assessing the pandemic’s impact on food insecurity, social connectivity, and mental health, as well as examining disparities in reported mortality and morbidity rates.

We identified five themes: (1) high COVID-19 morbidity and mortality rates, (2) increasing discrimination during COVID-19, (3) impact of COVID-19 on mental health, (4) difficulty in accessing healthcare, and (5) impact on SDoH. These themes were selected based on the summarized notes of key findings and details from each included article, as outlined in Table 1.

### 2.7. High COVID-19 Morbidity and Mortality Rates

Three documents reported on COVID-19 morbidity and mortality rates in racialized populations [26,32,36]. One study documented that Black, Arab, and South Asian individuals exhibited a higher incidence of exposure to COVID-19 [32]. One study examined the impact of COVID-19 on the health of the Black Canadian population and revealed notable disparities [26]. Mortality rates were elevated in Montreal neighborhoods with larger Black populations [26]. Morbidity rates were found to be higher among Black communities, with Black populations in Toronto experiencing a disproportionate 16% of hospitalizations despite comprising only 9% of the total population [26]. A study on COVID-19 prevalence in Montreal found that the five neighborhoods most affected by the virus had 1.4 times more residents identifying as visible minority [36].

The results from the grey literature sources indicated similar trends regarding COVID-19 impacts on racialized populations [42,43,44,45,46,54,57]. A report found that regions with 25% or more of visible minorities experienced disproportionately higher mortality rates compared to areas with fewer visible minorities particularly in Ontario, Quebec, and British Columbia, where mortality rates were over three times higher in visible minorities [44]. 

In the most racially diverse neighborhoods, COVID-19 infection rates were three times higher, hospitalization rates were four times higher, and death rates were twice as high compared to less diverse neighborhoods [46]. In Ontario specifically, White populations had the lowest rates of infection, while racialized populations experienced 1.2 to 7.1 times higher infection rates. Hospitalization rates were 1.7 to 9.1 times higher, and rates of critical illness were 2.1 to 10.4 times higher among racialized populations [43]. 

During COVID-19 waves 6 and 7, disparities in impacts based on neighborhood diversity diminished [42]. Infection rates among Black Torontonians reached 2400 per 100,000, with racialized individuals accounting for 77% of all COVID-19 cases and 79% of hospitalizations [54]. In Ottawa, racialized populations were also disproportionately represented among diagnosed cases, attributed to their prevalence in frontline jobs, and the lack of paid sick leave [45].

A study on reinfection rates revealed disparities among racialized groups, with Black Canadians experiencing higher frequency of reinfections, followed closely by individuals of Latin American and Chinese descent [57]. 

### 2.8. Increasing Discrimination During COVID-19

Seven documents [25,27,29,30,31,32,37] reported on stigmatization and racial discrimination. One study found that Asian Canadian students experienced discrimination on 25% of the days throughout the study, with Asian Canadians encountering direct, indirect, and vicarious discrimination on 10.5%/9.6% and 4.8% of their days, respectively [37]. Two documents identified elevated levels of COVID-19-related discrimination among Asian and Black individuals, highlighting persistent racism concerns expressed by Chinese Canadians [31,32]. 

Another study on Chinese Canadians revealed that second-generation participants were more apprehensive about employment discrimination, while first-generation individuals were more concerned about school bullying [30].

A study examining the connection between racial discrimination and adverse health behaviors found increased substance use, reduced sleep, and decreased exercise, particularly among Asian adults who associated discrimination with being perceived as carriers of COVID-19 [25]. One study indicated that individuals of East/South Asian were more likely to agree that racist attitudes had intensified in Canada, with South Asians expressing lower levels of comfort in seeking medical care [27]. 

Findings from the grey literature corroborated peer-reviewed articles [46,62,63,64]. Racialized populations reported heightened instances of harassment, racism, and stigmatization [46]. For example, 27% of respondents identifying as visible minorities felt unsafe when navigating their neighborhoods alone after dark [46]. This perceived escalation in harassment was pronounced among individuals of Chinese, Korean, and Southeast Asian descent [46]. Anti-Asian racism increased by 32%, with Southeast Asians witnessing a 121% rise in such incidences [62]. Assaults targeting individuals of Asian descent increased by 42% in 2021, while online hate incidents increased by 132% [62]. A report noted that Asian Canadians are often reluctant to discuss experiences of anti-Asian hate due to negative past experiences, doubts about the validity of their encounters, and pressure to adhere to the model minority stereotype [63]. This reluctance resulted in a range of negative emotions including fear, anger, helplessness, and frustration [63]. 

Chinese Canadians experienced high levels of racism during the pandemic [64]. Approximately 50% were subjected to name-calling or insults, while 30% frequently encountered racist graffiti or messages on social media [64]. Over half of Asian parents expressed concerns about their children being bullied due to their ethnicity, illustrating the pervasive impact of racism on families [64]. 

### 2.9. Impact of COVID-19 on Mental Health

Six documents examined the impact of COVID-19 on the mental health of racialized individuals [29,30,32,33,35,38]. One study found that resettled Syrian refugee women experienced exacerbated grief and anxiety, attributed to fears of contracting the virus and disrupted expectations of postnatal care due to stringent preventive measures [35]. In an ethno-culturally diverse sample from Québec, the pandemic’s impact on mental health varied by socioeconomic status and ethno-cultural background [32]. Individuals of Arab descent reported elevated psychological distress, while Black community members experienced heightened mental stress [32]. Among older racialized Muslim immigrants surveyed in Alberta, the majority (57.95%) reported no change in their mental health status before and during the pandemic, while approximately 36% indicated a deterioration in their mental health during this period [33]. A qualitative study conducted in the Greater Toronto Area (GTA) found that most participants experienced fear and anxiety related to COVID-19 during routine activities, particularly when accessing in-person healthcare services [38]. Racialized caregivers expressed anxiety about their dependents’ well-being if they (the caregiver) became ill [38]. Despite these challenges, many participants coped by drawing on self-reliance and community support. Over time, they adapted to the realities of the pandemic, with some even viewing the resulting lifestyle changes as a positive development [38]. 

In a study focusing on Chinese Canadians, first-generation individuals faced greater health, financial, and cultural pandemic-related threats, negatively impacting their mental health more than their second-generation counterparts [30]. For second-generation participants, financial strain was the only threat associated with poorer mental health [30]. Another study highlighted correlation between belief in the “Asian health hazard” stereotype and experiencing higher levels of distress and lower life satisfaction among East and Southeast Asians in both Canada and the US [29].

The negative mental health effects of the “Asian health hazard” were emphasized, linking this stereotype to increased distress and reduced life satisfaction among East and Southeast Asian Canadians [29]. Arab participants reported higher scores on The Hopkins Symptom Checklist-10 (HSCL-10)—a self-report questionnaire assessing depression and anxiety symptoms—and experienced greater pandemic-related mental health impacts compared to other racial and cultural groups, whereas East Asian participants reported lower scores [32]. Although COVID-19-related stigma did not significantly interact with symptoms overall, higher stigma was linked to worse mental health among South Asian and Black participants [32]. For White participants, both exposure to the virus and COVID-19-related discrimination were significantly associated with poorer mental health [32]. 

The grey literature reported that racialized populations experienced significant negative mental health impacts [51,52,55,60]. A report on racialized communities in the GTA, indicated substantial adversities in mental health during the first three waves of the pandemic, with social isolation cited as the primary stressor [51]. 

A study on Canadian Arab populations found that newcomers faced challenges due to a lack of social networks, with 77% experiencing increased stress during the pandemic [52].

A survey examining the impact of COVID-19 on sexually transmitted and blood-borne infections (STBBI)-related health services for African, Caribbean, and Black (ABC) individuals in Canada revealed that 33% reported worsened mental health since the pandemic’s onset attributed to challenges in accessing mental health services [55]. 

A report on Indigenous populations highlighted emerging mental health challenges due to restricted access to cultural practices, heightened fear of contracting the virus, and the inability to travel or engage in recreational activities [60]. 

### 2.10. Difficulty in Accessing Healthcare

Three documents examined the challenges faced by racialized communities in accessing healthcare [33,35,38]. A study highlighted the barriers faced by resettled Syrian refugee women in accessing postnatal healthcare services, emphasizing how restrictions on in-hospital support, limited childcare options, the transition to virtual care, and reduced access to interpretation services exacerbated their challenges and feelings of isolation during childbirth [35]. Another study reported unmet healthcare needs among older racialized Albertans, with 3.4% of the sampled population indicating gaps in medical and health service support [33]. Disruptions across the healthcare sector led to significant service interruptions, including in dentistry and ophthalmology, increased wait times, delayed healthcare-seeking behavior until conditions became acute or prolonged, and concerns about the effectiveness of virtual healthcare services in providing accurate diagnosis and treatment [38]. 

The grey literature had similar findings to those of peer-reviewed articles [45,55,59,60,61]. One report indicated additional barriers faced by racialized communities, such as a lack of family doctors, and limited awareness of available supports services [45]. 

A report focusing on ABC communities found that 38% of participants living with HIV encountered challenges in accessing care providers or clinics since the onset of the pandemic [55]. 

During the first year of the pandemic, First Nations, Inuit, and Métis individuals, especially those living off-reserve, faced disproportionate challenges in accessing healthcare services, with nearly half reporting difficulties in scheduling appointments and significant barriers to mental health and addiction therapy, as well as consultations with medical specialists [59]. Another report highlighted that First Nations communities, particularly in remote areas, faced inadequate access to healthcare and emergency services, compounded by the reliance on air transportation for specialized medical care and the lack of reliable, sustainable water systems that hindered basic preventive measures like handwashing [61]. Structural inequalities, including insufficient access to healthcare services, heightened the vulnerability of Indigenous communities to the adverse effects of the pandemic [60]. 

### 2.11. Impact on the SDoH

Five documents reported on the impact that COVID-19 had on the SDoH of racialized communities [28,33,34,35,38]. Findings from the grey literature also spoke to the economic and social impact of the pandemic on racialized communities [39,40,41,47,48,49,50,53,55,56,58,61]. Amongst the reported impacts were childcare shortages [35,39,41], food insecurity [28,33,34], and social isolation [33,38]. Economic disruptions disproportionately affected racialized populations due to overrepresentation in precarious sectors, inadequate policy supports, and systemic inequities, especially among Indigenous, Black, Arab, and migrant populations [47,48,49,52,58,65]. 

### 2.12. The Childcare Dilemma and Unemployment Struggles

One study highlighted that Syrian refugee women faced heightened childcare responsibilities during COVID-19 due to the loss of informal support networks who were unable to help during childbirth experiences [35]. 

The childcare crisis, as discussed in two grey literature reports, has become a burden to parents looking for a job due to the scarcity of space and the accompanying costs [39,41]. Immigrant women without support systems face heightened economic insecurity and limited socioeconomic opportunities due to a lack of affordable childcare [39,41]. Statistics reveal that the unemployment rate for women of South Asian descent in Canada soared to nearly double the rate of the general population in July 2020, registering at 20.4% compared to the national average of 11.3% [41]. 

### 2.13. Challenges with Accessing Food

One study that recruited older Albertan immigrants disclosed that 2.2% of participants missed supports in accessing nutritious food, while 5.6% lacked assistance with grocery shopping [33]. Another study reporting on the experiences of urban Indigenous people living in Saskatchewan revealed that participants encountered exacerbated food insecurity and access challenges during the pandemic [34]. The study revealed significant food insecurity, with 33.3% experiencing severe, 22.8% moderate, and 12.3% marginal levels [34]. Post-pandemic, 47.1% struggled with rising food costs, 31.7% faced restricted supermarket access, and 41.4% noted limited food availability [34]. Income loss due to the pandemic was reported by 25% [34]. 

### 2.14. Coping with Challenges with Accessing Food

To cope, older Albertan immigrants relied on community resources, adjusted eating habits, sought assistance from family and friends, meal-planned, gardened, searched for deals, explored alternative protein sources, and prioritized children’s nutrition [34]. A study on the impact of COVID-19 on food security in Nunavut highlighted the importance of funding programs in improving community food access [28]. The participants stressed that achieving food sovereignty requires decolonization and support for locally harvested foods to strengthen the food economy [28]. They also emphasized the need for community-driven efforts to assist residents during crises and address gaps in support systems [28]. 

### 2.15. Feelings of Loneliness and Impact on Social Networks

Amongst racialized Muslim Albertans, 44.32% suffered from loneliness, due to factors like reduced family contact and social exclusion [33]. Most participants (93.18%) preferred in-person events over virtual programming, suggesting virtual options did not meet their social needs [33]. The study highlighted barriers to connectivity, such as technological issues and limited transportation [33]. Additionally, 11.3% lacked affordable housing support and reported reduced community assistance during lockdowns due to funding and resource cuts [33]. A study on predominantly racialized refugees and immigrants in the GTA found that all participants relied on social networks for information about financial subsidies and health awareness [38]. Around one-third faced difficulties accessing food delivery services and personal protective equipment, due to unawareness or resource scarcity [38]. Many expressed frustration with technological issues and disruptions in accessing support systems [38]. The participants noted a decline in social interactions but emphasized their acceptance and tolerance of the changes brought by the pandemic [38]. 

### 2.16. Economic Impacts

A report published six months into the pandemic revealed that COVID-19’s economic impact disproportionately affected racialized individuals who are overrepresented in sectors like food and accommodation services, as these sectors had to shut down, operate at reduced hours or capacity, which lead to many individuals losing their jobs [47]. Another report found that 45% of Canadian Arab respondents reported a negative impact on their finances, with a quarter having experienced reduced work hours or job loss attributed to the pandemic [52]. One study highlighted that Black, Indigenous and racialized individuals in Canada shouldered a disproportionate burden of economic insecurity stemming from pandemic-induced job losses [65]. 

A report covering economic insecurity trends between July 2020 and June 2021 showed that 31% of racialized individuals and 28% of Indigenous individuals faced economic insecurity [61]. It highlighted a growing unemployment gap between racialized and non-racialized workers, partly due to racialized workers being more likely to work in sectors like accommodation and food services, information, culture, recreation, and wholesale and retail trade—industries that accounted for 80% of job losses during the pandemic [61]. Indigenous peoples have experienced significant economic setbacks, characterized by elevated rates of unemployment and financial strain in comparison to non-Indigenous populations [47]. A report on the labor market impacts of COVID-19 on Indigenous people living off-reserve found that Indigenous individuals experienced a slower recovery in employment compared to non-Indigenous people [58]. In regions like British Columbia and Alberta, initial employment declines were significant, and although employment rates increased afterward, they remained below pre-pandemic levels [58]. The recovery was particularly slow in occupations that traditionally employed many Indigenous workers [58]. 

Black Canadians faced higher rates of layoffs and financial stress, with significant worries about rent payments [53]. A report highlighted the economic challenges faced by Black-led households during the pandemic, noting that from July 2020 to June 2021, the unemployment rate for Black individuals aged 15 to 69 averaged 12.9%, significantly higher than the 7.9% rate for the rest of the non-racialized population [56]. 

A survey-based report from ABC communities revealed significant economic impacts, with 36% of respondents facing reduced hours, pay cuts, or job loss, while 61% reported difficulties paying bills, and 53% experienced food insecurity [55].

### 2.17. Essential Workers and Job-Nature-Related Impacts

A report on hotel workers highlighted the severe effects of pandemic-related closures on the hospitality sector, which relies heavily on lower-paid racialized and migrant workers, who faced heightened health and safety risks, including increased exposure to COVID-19 at work, while the widespread closures led to massive job losses, worsening income insecurity and stress [48]. 

A report on migrant workers, mostly racialized individuals, revealed significant challenges due to their lack of permanent resident status, which hindered their ability to assert rights, access healthcare services, and secure suitable housing—essential for sheltering from the virus and practicing self-isolation [49]. Another report highlighted a troubling rise in wage theft and significant financial repercussions for migrant workers due to border closures, with quarantine requirements failing to meet their needs, resulting in unpaid quarantine periods and limited access to food [50]. 

## 3. Discussion

Health inequities between racialized and non-racialized (White) populations have persisted long before the onset of the COVID-19 pandemic; therefore, it was anticipated that the pandemic would exacerbate these existing disparities [66]. 

A scoping review methodology was selected for this study to facilitate a comprehensive examination of heterogeneous data, which are well-suited to the broad scope of our research objective—namely, to investigate the impact of COVID-19 on health, mortality and morbidity, and the social determinants of health among racialized populations. The use of a narrative synthesis approach to integrate the findings was deemed appropriate, given its suitability for synthesizing diverse and methodologically varied evidence.

Racialized individuals have faced worsened mental health issues, limited access to healthcare, discrimination, job loss, severe economic impacts, and higher rates of morbidity and mortality from COVID-19 compared to non-racialized populations. Additionally, our findings report that COVID-19 disproportionately impacted several SDoH areas including childcare challenges, food insecurity, social isolation, and economic instability. Social networks, crucial for support and information, were disrupted by lockdowns and technological barriers, intensifying loneliness and exclusion. Economically, racialized workers in hard-hit sectors faced greater job losses, slower recovery, and financial stress, with migrant workers experiencing additional vulnerabilities due to precarious status and inadequate protections.

Our findings are consistent with the existing literature, which reports similar impacts in other high-income countries, highlighting common challenges faced by racialized populations [67,68,69]. Data from the United States, the United Kingdom, Sweden, and the Netherlands reveal ethnic inequalities in COVID-19 risks and outcome, which might be attributed in part to institutional racism and discrimination [67,68,69]. These findings suggest that COVID-19 may function as a social disease, with its impact differing based on an individual’s socioeconomic position [70]. 

Our findings indicate that the impact of the pandemic on racialized groups may have been magnified by compounding factors, such as employment in low-wage occupations, being a new immigrant or migrant worker, residing in rural areas, and having minor dependents, all of which likely worsened the pandemic’s effects on these communities [30,32,33,34,35,38,39,40,41,45,46,47,48,50,51,53,56,58,59,60,61]. 

Responsive policy action is crucial to mitigate the compounding factors that have exacerbated the negative effects of the COVID-19 pandemic on racialized populations. For example, the lack of universal paid sick leave policies has left low-income racialized populations in a difficult position, forcing them to work while ill [45]. Paid sick leave has been linked to improved mental and self-rated health outcomes and a reduction in the spread of airborne pathogens [71,72]. Another important area for legislative intervention is the implementation of strict anti-racism policies that address covert racism and discriminatory practices in healthcare and public spaces. Racism and discrimination have been shown to reduce trust in the healthcare system, delay healthcare-seeking behaviors, [73,74] and contribute to self-blame [63]. These measures are essential for recovering from the pandemic’s impact, preventing future crises, and ensuring equitable healthcare access and support services for all.

A notable paradox found in our findings was that East Asian participants reported lower HSCL scores despite experiencing disproportionately high levels of COVID-19-related discrimination [32]. This phenomenon may reflect cultural differences in the expression of psychological distress, with East Asians potentially less inclined to report such symptoms compared to other groups [32]. Conversely, among White participants, discrimination correlated with elevated HSCL-10 scores, suggesting greater psychological vulnerability potentially linked to their majority group status and corresponding expectations of social protection [32]. These findings underscore differential impacts of pandemic-related discrimination on mental health across cultural groups and emphasize the importance of addressing biases inherent in self-reported data. Moreover, they call for the development of culturally sensitive assessment tools that better account for normative variations in distress expression.

## 4. Future Recommendations

Addressing the intersecting challenges faced by racialized populations requires holistic and multi-level interventions aimed at dismantling systemic inequities and promoting health equity across all sectors of society. Legislative action should prioritize the implementation of robust anti-racism policies that target both overt and covert forms of discrimination within healthcare systems and public spaces. Future research should be directed toward the development and evaluation of interventions that specifically address the disproportionate burdens borne by racialized communities. Longitudinal studies are particularly critical to understanding the enduring impacts of the pandemic, especially in relation to widening socioeconomic inequalities. Furthermore, the establishment of a centralized, standardized database for the systematic collection of race-based data is essential. Such a resource would enable more accurate tracking of disease burden, facilitate a deeper understanding of structural disparities, and inform the design of targeted, evidence-based public health responses.

## 5. Limitations

This review is limited by the scope of the search strategy and the inclusion of grey literature, which may vary in quality and lack peer review. The heterogeneity of the included studies may have introduced measurement, selection, and information biases, potentially affecting the generalizability of the findings. We acknowledge that the results do not capture the experiences of frontline workers or individuals living with comorbidities, further limiting their generalizability. These groups were excluded due to their heightened exposure risks or underlying health conditions, which could confound the observed impacts. While we acknowledge that structural inequities contribute to the increased susceptibility of some racialized groups to comorbidities, the primary objective of this review was to examine the broad impact of COVID-19 on racialized populations. Including the above compounding factors would require a broader analytical scope to fully capture their influence—an undertaking beyond the parameters of the current study. We aimed to provide a comprehensive account of the impacts of COVID-19 on racialized populations, including the identification of coping strategies related to food insecurity. Nonetheless, a key limitation lies in the limited exploration of coping mechanisms addressing financial, health, and social challenges. This gap reflects this study’s primary focus on the direct impacts of the pandemic, rather than the broader spectrum of adaptive responses.

## 6. Conclusions

COVID-19 has exacerbated existing disparities, disproportionately affecting racialized groups and underscoring the urgent need for equity-focused policies and preparedness strategies. To prevent these communities from bearing unequal burdens in future health crises, we recommend the implementation of anti-racism policies and mandated paid sick leave. Additionally, we call for the establishment of a centralized database to systematically collect race-based data. This system would help track disease burden, identify gaps, and monitor racism and discrimination in healthcare, ultimately guiding data-driven actions and the development of targeted interventions.

## Figures and Tables

**Table 1:** Included peer-reviewed articles’ characteristics (*n* = 15).

References	Country	Study Design	Population	Objectives
[25]	Canada	Quantitative	Racialized adults in Alberta	To examine the differential impacts of racial and non-racial discrimination on health behavior change in the first wave of the pandemic, as compared to visible minority adults who did not experience discrimination during this period.
[26]	Canada	Systematic review	Black populations across Canada	To identify the health impact of COVID-19 on mortality, morbidity, hospital admission, and hospital readmission rates in the Black population across Canada.
[27]	Canada	Quantitative	Ontario residents	To explore perceptions and experiences of stigma among Ontario residents by gender, age, and race/ethnicity.
[28]	Canada	Qualitative	Community members from Iqaluit and Arviat (Nunavut)	To document the impacts of COVID-19 outbreaks and associated funding programs, and to explore community perspectives on these impacts to provide insights into how food security and food sovereignty may be better supported in Nunavut in the future.
[28]	Canada	Qualitative	Immigrants and refugees residing in Toronto or the Greater Toronto Area, Ontario	To explore how immigrants and refugees experienced access to health and social services during the first wave of COVID-19 in Toronto, Canada.
[29]	US and Canada	Mixed methods	Adult Asian Americans and Asian Canadians	To measure the content of the Asian health hazard stereotype, and to investigate the association between beliefs that this stereotype is held by others and psychological well-being for East and Southeast Asian Americans and Canadians specifically (but not exclusively) during the COVID-19 pandemic.
[30]	Canada	Quantitative	Chinese Canadian (CC) adults	To examine how the rise in anti-Chinese discrimination, in addition to the threats posed by the COVID-19, have affected not only CCs’ well-being, but also their Chinese and Canadian identities.
[31]	Canada	Quantitative	Chinese Canadian (CC) adults	To investigate (a) whether perceived personal and group discrimination make distinct contributions to CCs negative affect and concern that the heightened discrimination they experienced during the pandemic will continue after the pandemic; (b) whether Canadian and Chinese identities and social support moderate the effect of discrimination on this concern; and (c) whether race-based rejection sensitivity (RS) explains why each type of discrimination predicts negative affect and expectation of future discrimination.
[32]	Canada	Quantitative	A culturally diverse sample of adults in Quebec	To investigate the association of exposure to the virus, COVID-19-related discrimination, and stigma with mental health during the COVID-19 pandemic, in a culturally diverse sample of adults in Quebec (Canada).
[33]	Canada	Mixed methods	Muslim older immigrants aged 50+, living in Edmonton, Alberta	To explore the experiences of Muslim older adults during the COVID-19 pandemic to identify ways to build community resilience as part of a community-based participatory research partnership with a mosque in Edmonton, Alberta.
[34]	Canada	Mixed methods	Indigenous (including First Nations and Métis) adults living in urban areas of Saskatchewan	To examine the impact of the pandemic and related lockdown measures on the food security of urban Indigenous peoples in Saskatchewan, Canada.
[35]	Canada	Qualitative	Postnatal re-settled Syrian refugee women in Nova Scotia with a child under 12 months of age	To understand Syrian refugee women’s experiences with accessing postnatal healthcare services and supports during the COVID-19 pandemic.
[36]	Canada	Quantitative	Residents of 15 neighborhoods in Montreal, Quebec	To examine the social ecology of COVID-19 risk exposure across Montreal by comparing fifteen neighborhoods with contrasting COVID-19 prevalence.
[37]	US and Canada	Mixed methods	Asian American and Asian Canadian students	To explore if self-compassion was associated with subjective well-being and protective behaviors for American and Canadian Asians who faced COVID-19 discrimination.

**Table 2:** Results from peer-reviewed articles (n = 15).

References	Sample Size, N: Sample of Interest *, n: Race/Ethnic Composition	Findings
[25]	N = 986 *; n = 210 * Researchers excluded the data for those who identified as Caucasian. (Race/ethnic composition of (n): Asian (54.8%), Indigenous (10.5%), African American (9.0%), Middle Eastern (6.2%), Latino (4.3%), and mixed race/ethnicity (17.6%))	A total of 56.2% of the population examined experienced discrimination in the previous month; 26.7% reported it was race-related. Asian adults reported more racial discrimination and discrimination due to people believing they had COVID-19 compared to other visible minorities. Racial discrimination during the pandemic was strongly associated with increased substance use (OR: 4.0) and decreased sleep (OR: 7.0) and was weakly associated with decreased exercise (OR: 2.2). Non-racial discrimination was strongly associated with decreased sleep (OR: 4.8).
[26]	N/A	Mortality rate: in Montreal neighborhoods with higher shares of Black residents, the mortality rate due to COVID-19 was notably higher. Morbidity rate: higher morbidity rates in Black populations compared to other racial groups. Hospital admission rate: in Toronto, Black individuals made up 16% of hospitalizations but only 9% of population, indicating a higher hospital admission rate compared to other racial groups.
[27]	N = 1823; n = 1227 (Racial/ethnic composition of (N): 33% White, 26% East/Southeast Asian, 14% Black, 12% South Asian).	A total of 51% of participants agreed/strongly agreed that racist views had increased toward certain racial/ethnic groups in Canada during the pandemic. Perceived stigmatization during the pandemic included race/ethnicity (37%), political beliefs (26%), older age (24%), being a healthcare worker (23%), younger age (22%), being an essential worker (21%), and gender (11%). In total, 39% feared experiencing and 37% experienced stigmatization during the pandemic, with men, individuals aged 18–40, and racialized participants more likely to fear or experience stigma. Black participants were less likely to indicate comfort with masking in public, seeking medical care, and being tested for COVID-19. South Asian participants were also less likely to be comfortable seeking medical care.
[28]	N = 7; n = 7 (Racial/ethnic composition: 100% Indigenous)	Key themes included the following: Decolonization is crucial for achieving food sovereignty. Food sharing plays a vital role in community cohesion. Communities exhibited resilience during the COVID-19 pandemic. There is a desire for greater support and strengthening of locally harvested food economies. Increasing knowledge of food and harvesting skills is seen as essential. Efforts are needed to reach residents who may be overlooked during times of need or crisis.
[38]	N = 72; n = 72 (Country of origin (N) by %: Pakistan: 15.3%, China: 15.3%, Bangladesh: 9.7%, Afghanistan 6.9%, South Korea: 6.9%, India: 6.9%, Syria: 5.6%, Philippines: 5.6%, other: 27.8%	Most participants experienced fear and anxiety during the COVID-19 outbreak. However, many coped through a combination of self-reliance and community support. Over time, they came to terms with the realities of the pandemic. Some individuals even found the lifestyle changes brought about by the pandemic to be a positive experience.
[29]	N = 703; n = 351 * * Participants who identified as Chinese Canadians	Belief in the Asian health hazard stereotype correlated with higher distress and lower life satisfaction for East and Southeast Asians in both Canada and the U.S. No significant differences observed between East and Southeast Asian Americans and Canadians, as well as between Chinese and non-Chinese participants. These effects remained robust even when considering pandemic- and discrimination-related stressors. Asian health hazard and perpetual foreigner stereotypes were shown to be psychometrically distinct.
[30]	N = 874; n = 874 (Racial/ethnic composition: Chinese Canadian 100%, first generation (G1) 71.9%, second generation (G2) 28.1%)	Respondents experienced less respect due to ethnicity G1: 60.6%; G2: 56.8%. Reported personal threats or intimidation: G1: 35.2%; G2: 39.8%. Generational status influenced pandemic experiences: G1 perceived more health, financial, and cultural threats; G2 reported more personal and group discrimination. Perceived discrimination correlated with negative effects. The type of discrimination affected cultural identities: personal discrimination linked to lower Canadian identity; group discrimination linked to stronger Chinese identity. Only about 10% of those harassed reported encounters to authorities or on social media. Perceptions of being seen as perpetual foreigners hindered harassment reporting.
[31]	N = 516; n = 516 (Racial/ethnic composition: Chinese Canadian 100%)	Personal and group discrimination positively correlated and were associated with negative emotion and expectation of future discrimination. CC individuals with stronger identification as Chinese experienced less adverse impact from group discrimination. Stronger identification as Canadian associated with greater impact from personal discrimination. Path analysis showed both types of discrimination positively related to racial sensitivity (RS). RS predicted expectation of continued racism post-pandemic.
[32]	N = 3273; n = 1667 (Racial/ethnic composition of (N): 49.07% White, 7.61% East Asian, 2.93% South Asian, 21.14% Black, 3.64% Southeast Asian, 13.75% Arab, 1.86% Other)	Mental health varied significantly based on socioeconomic status and ethno-cultural group, with those with lower incomes and Arab participants reporting higher psychological distress. Exposure to the virus, COVID-19-related discrimination, and stigma were associated with poorer mental health. Associations with mental health varied across ethno-cultural groups, with exposed and discriminated Black participants reporting higher mental distress.
[33]	N = 104; n = 104 (Place of birth: 14.7% Ethiopia, 28.4% Lebanon, 19.3% Somalia, 37.5% Other)	Mental health: 57.95% reported the same mental health status before and during the pandemic. A total of 32 respondents reported worse mental health during the pandemic. Physical health: 54.55% reported no change during the pandemic; however, 38 respondents reported worse physical health status after the pandemic started. Loneliness: the study identified the theme of “Triple jeopardy leads to loneliness”, with COVID-19-related reasons of lack of contact with family, lack of community connectivity via local mosques, and social exclusion leading to loneliness. Food security: 2.2% reported missing support in nutritious food; 5.6% reported missing supports in grocery shopping. Housing security: 11.3% reported missing support in affordable housing. Healthcare access: 3.4% reported missing support in medical/health services.
[34]	N = 130; n = 130 (Racial/ethnic composition: 100% Indigenous)	Food insecurity/access: during the pandemic, 33.3% experienced severe food insecurity. Food expenses increased for many households after the onset of COVID-19. Coping strategies to deal with food access challenges included the following: relying on community resources and government food distribution programs, (40.7%), changing eating habits (33.3%), turning to family and friends for financial and food support (27.8%), and adopting budgeting and meal planning strategies (24.1%). Income: 25.0% of respondents experienced pandemic-related income losses.
[35]	N = 8; n = 8 (Racial/ethnic composition: 100% Syrian refugee)	Anxiety and grief theme: “fear of COVID-19” and “broken expectations” for postnatal care experiences. Social support theme: loss of informal support, which encompassed topics of “missing support people” and greater “childcare burden” during COVID-19. Inequitable healthcare access theme: limited postnatal healthcare services with regards to in-hospital support people, childcare restrictions, changes to services delivery, and moves towards virtual care. This resulted in isolated birthing experiences, as well as challenges with accessing interpretation services.
[36]	N = 1,704,694 (15 neighborhoods), Visible minority: 34.2%	The five Montreal neighborhoods most affected by the COVID-19 pandemic had a share of residents that belonged to a visible minority 1.4 times greater (30.6%; 21.4%).
[37]	N = 127; n = 42 (Racial/ethnic composition of (n): 100% Asian Canadian)	Discrimination experienced on 25% of days (over the entire study). Asian Canadians: direct, indirect, or vicarious discrimination experience on 10.5%, 9.6% and 4.8% of their days, respectively.

* Sample of interest: individuals who identified as racialized.

**Table 3:** The results from the grey literature—grouped by population (n = 24).

Racialized Populations (General)	
[39]	**Employment and Income:** Overrepresented in low-wage jobs and most economically disrupted; higher workplace exposure risk linked to mortality disparities.
[40]	**Gender and Race Wage Gaps:** Racialized and immigrant women experienced the lowest earnings, especially in care work.
[41]	**Employment:** Racialized women were more severely affected by job losses (e.g., South Asian women had 20.4% unemployment vs. 11.3% national rate). **Childcare Access:** Limited access to affordable childcare hindered workforce participation.
[42]	**Infection Disparities:** Most racially diverse neighborhoods had 1.6× higher infection, 2.0× higher hospitalization/ICU rates, and 2.6× higher death rates.
[43]	**Infection Disparities:** racialized Ontarians had 1.2–7.1× higher infection and 1.7–7.6× higher death rates compared to white Ontarians.
[42]	**Higher Infection and Severity in Diverse Neighborhoods:** The age-adjusted COVID-19 incidence rate was 1.6 times higher in the most racially diverse neighborhoods compared to the least diverse. Severe outcomes were also significantly higher: Hospitalization and ICU admission rates were 2.0 times higher. Death rates were 2.6 times higher. **Persistent Disparities:** while recent data show fewer differences based on neighborhood diversity, the most racially diverse neighborhoods have consistently experienced a disproportionate burden of severe COVID-19 outcomes throughout the pandemic.
[44]	**Higher COVID-19 Mortality in Racialized Areas:** areas where visible minorities make up 25% or more of the population had COVID-19 mortality rates about twice as high as areas with less than 1% visible minority population. **Urban Impact:** these disparities were largely concentrated in Canada’s largest cities: In Montreal, areas with the highest proportion of Black Canadians had a mortality rate of 149.3 per 100,000, compared to 88.1 per 100,000 in areas with the lowest. In Toronto, the difference was smaller among South Asian populations: 26.2 vs. 35 per 100,000. **Ongoing Socioeconomic Disparities:** Visible minority groups continue to face higher unemployment, more financial hardship, and greater representation in low-wage work.
[45]	**Disproportionate COVID-19 Impact:** racialized groups in Ottawa were overrepresented among those testing positive for COVID-19, according to early data from June 2020. **Systemic Barriers:** systemic racism and discrimination limited access to stable employment, placing many racialized individuals in high-risk frontline roles (e.g., cleaners, ride-share drivers). **Workplace and Transit Risks:** Lack of paid sick leave forced many to choose between income and health. Dependence on public transit increased exposure, hindered testing access, and contributed to food insecurity and social isolation. **Economic and Mental Health Impacts:** Pandemic-related job losses worsened financial and food insecurity. Mental health was negatively affected by stressors such as financial instability, isolation, over-policing, and reduced access to counseling. **Barriers to Healthcare:** Challenges included lack of access to family doctors and limited awareness of available supports. **Inequitable Lockdown Effects:** Lockdowns disproportionately affected racialized communities due to limited living space and uneven enforcement of public health bylaws.
[46]	**Frontline Workforce Overrepresentation:** A total of 34% of frontline/essential workers identified as visible minorities (vs. 21% in other sectors) pre-COVID-19. In July 2020, 24% of employed Filipino Canadians and 20% of Black Canadians worked in this sector, compared to 14% of all workers. **Racial Discrimination and Harassment:** visible minority respondents were three times more likely to report increased harassment or attacks based on race, ethnicity, or skin color (18% vs. 6%). **Health Disparities by Neighborhood Diversity (Ontario):** COVID-19 impacts were significantly worse in the most racially diverse neighborhoods: 3× higher infection rates. 4× higher hospitalization rates. 2× higher death rates compared to the least diverse neighborhoods.
[47]	**Disproportionate Job Loss:** Visible minorities were overrepresented in sectors hardest hit by COVID-19 (e.g., food and accommodation), leading to higher unemployment. In August 2020, unemployment for selected visible minority groups ranged from 12.7% to 17.9%, compared to 9.4% for the non-Indigenous, non-visible minority population.
[48]	**Hotel workers:** hotel cleaning staff are typically lower-paid and predominantly female, racialized, immigrant, or migrant workers, reflecting a highly segmented workforce. **Occupational Health and Safety Risks:** On-site work requirements increased the risk of COVID-19 exposure. Long commutes on public transit to downtown Toronto hotels added to this risk. Workers often felt they had no choice but to continue working despite fears, due to financial necessity. Those not currently employed expressed anxiety about returning, raising concerns about future staff retention and re-hiring. **Health Impacts:** workers reported significant worry and fear about infection, especially among those who are older, have health conditions, or care for families—highlighting both physical and mental health concerns.
[49]	**Foreign Workers:** temporary foreign workers experienced outbreaks despite exemptions; at least 3 deaths occurred.
[50]	**Precarious Immigration Status:** lack of permanent resident status limited migrant workers’ ability to assert their rights, protect themselves from COVID-19, access healthcare, and secure adequate housing. **Wage Theft and Exploitation:** wage theft was widespread, worsened by border closures that caused income loss and pressured workers to enter Canada without sufficient support. **Inadequate Quarantine Measures:** quarantine protocols failed to address migrant workers’ needs, leading to unpaid isolation periods, food insecurity, and restricted mobility. **Discrimination and Abuse:** reports of intimidation, surveillance, threats, and racism increased—particularly targeting Caribbean workers. **Legal Exclusion and Overwork:** existing labor laws excluded many migrant workers, leaving them vulnerable to exploitation and intensified workloads.
[51]	**Mental Health Impacts During COVID-19:** **Worsening Mental Health:** Participants faced significant social isolation and stress, negatively affecting their well-being. **Barriers to Support:** Access to mental health services was limited by financial issues, virtual service challenges, stigma, language and cultural barriers, and mistrust in institutions. **Influence of Racism and Discrimination:** Experiences of racism and discrimination played a critical role in shaping mental health outcomes. **Socioeconomic Stressors:** Factors such as job loss, economic and housing insecurity, and caregiving responsibilities intensified mental health challenges, especially when combined with pandemic-specific pressures. **Structural Inequities:** Systemic racism contributed to greater exposure to health risks and stress among racialized groups, compounding mental health burdens.
**Arab Canadians**	
[52]	**Economic Impact:** Arab respondents were somewhat less affected economically by the COVID-19 pandemic compared to non-Arabs: 25% reported reduced hours or job loss (vs. 33% of non-Arabs). 45% reported an overall negative financial impact (vs. 52%). 14% requested payment deferrals from banks (vs. 20%). **Mental Health Impact:** 77% of Arab respondents reported increased stress levels, slightly lower than the 81% reported by non-Arabs.
[46]	In August 2020, unemployment was highest among Arab Canadians (17.9%), while the lowest was non-visible minority or Indigenous Canadians (9.4%).
**Black/African/Caribbean Canadians**	
[53]	**Health and Financial Impact:** Faced higher rates of COVID-19 symptoms, hospitalizations, and deaths; more likely to hold frontline jobs and experience income loss and housing insecurity.
[54]	**COVID-19 infections and mortality:** Black Torontonians experienced COVID-19 infection rates exceeding 2400 per 100,000. Racialized groups represented 77% of all cases and 79% of hospitalizations in Toronto. During the first wave, Black communities were disproportionately impacted, accounting for 33% of monthly case rates at their peak in August 2020. **Systemic inequities in racialized neighborhoods**—such as overcrowded housing, precarious employment, and barriers to healthcare—undermined the effectiveness of public health measures, especially in the second wave.
[55]	**Mental Health:** 33% reported worsened mental health; 42% attempted to access mental health services, with 62% experiencing barriers
[55]	**Substance Use:** increased use of alcohol (38%), cannabis (56%), and other substances reported; low engagement with support services
[55]	**Food and Financial Insecurity**: 61% reported financial hardship; 53% faced food insecurity since the pandemic began.
[56]	**Economic Hardship:** 40% of Black-led households experienced economic hardship during the pandemic, with 75% attributing it directly to COVID-19. **Higher Unemployment:** from July 2020 to June 2021, the average unemployment rate for Black individuals aged 15 to 69 was 12.9%, significantly higher than the 7.9% rate among non-racialized populations.
[57]	**Reinfection:** Black (30.3%) Canadians more frequently reported having multiple infections than Canadians with Latin American (21.7%), Chinese (18.3%), Filipino (17.9%), Arab (12.1%) and West Asian (9.1%) backgrounds.
**Indigenous Populations**	
[58]	**Employment:** Employment recovery lagged behind non-Indigenous populations; higher unemployment as of August 2021. While most occupation groups saw net employment gains in the most recent six-month period, growth was weaker in those occupations that are most common among Indigenous workers compared to the same period in 2019.
[47]	**Greater Financial Strain:** 36% of Indigenous respondents reported difficulty meeting financial obligations, versus 25% of non-Indigenous participants.
[59]	**Healthcare Access:** First Nations, Métis, and Inuit people reported more unmet healthcare needs and service disruptions than non-Indigenous people.
[60]	**Community Strength and Challenges:** Highlighted community resilience, but systemic issues (housing, water access, internet, health services) exacerbated pandemic vulnerabilities.
[60]	**Mental Health and Substance Use:** Concerns about reduced access to cultural practices and misuse of CERB funds were raised. Multiple participants brought forward concerns around some individuals who used the Canada Emergency Response Benefit (CERB) to purchase substances (e.g., drugs and/or alcohol).
[61]	**Limited Healthcare Infrastructure:** Most First Nations communities in isolated or remote areas have small healthcare facilities. Those needing acute COVID-19 care, such as ventilators, must be airlifted, causing logistical challenges due to limited availability of planes and helicopters. **Inadequate Isolation Facilities:** Isolation options for sick individuals on reserves were limited. **Double Jeopardy:** First Nations people faced higher health risks but had less access to necessary healthcare services. **Housing Challenges:** Poor quality, inadequate, and unsafe housing on reserves increased COVID-19 exposure and vulnerability for First Nations populations.
**South Asian, Southeast Asian, and Chinese Canadians**	
[62]	**Anti-Asian Racism**: Incidents rose 32% in 2021. South Asian and Southeast Asian reports rose 318% and 121% respectively. Racist physical assaults rose 42%; online hate incidents rose 132%. Nearly half of incidents occurred in public spaces.
[63]	**Chinese Canadians:** Experienced widespread anti-Asian racism, often unreported due to internalized doubt and social pressures.
[64]	**Widespread Experiences of Racism:** In total, 50% of the respondents reported being called names or insulted due to COVID-19; 43% faced threats or intimidation. In total, 30% of the respondents encountered racist graffiti or social media messages, and 29% frequently felt they were viewed as a threat to others’ health. **Perceived Blame and Belonging:** Most believed Canadians blamed people of Chinese ethnicity for the pandemic. Only 13% felt they were always perceived as fully Canadian. **Impact on Daily Life and Safety:** In total, 61% changed their routines to avoid potential racism or danger. In total, 64% felt North American media contributed to negative views of Chinese people. **Concerns for Children:** Over half were concerned Asian children would face bullying when returning to school.
[46]	**High Exposure and Job Loss Risk:** Visible minorities were more concentrated in hard-hit sectors like food and accommodation—especially Korean (19.1%), Filipino (14.2%), and Southeast Asian (14.0%) Canadians.
[47]	**Rising Harassment and Stigma:** Visible minorities, particularly Chinese, Korean, and Southeast Asian individuals, reported increased harassment and stigma. Visible minority respondents were three times more likely than others to perceive an increase in race-based harassment or attacks.

## Data Availability

The data from which the findings are generated are available upon reasonable request to the corresponding author.

## Supplementary Materials

The following supporting information can be downloaded at: https://www.mdpi.com/article/10.3390/ijerph22071054/s1, Supplementary File S1: PRISMA-ScR Checklist.

## Appendix A. Peer-Reviewed Articles’ Search Strategy

The following search strategy was copied to all of the included databases when appropriate.

Ovid MEDLINE

1  exp pneumonia, viral/or exp coronavirus infections/
2  exp Racial Groups/
3  exp “American Indian or Alaska Native”/
4  exp Indians, North American/
5  middle eastern canadians.mp.
6  exp “Health Disparate, Minority and Vulnerable Populations”/
7  exp social problems/or exp socioeconomic factors/
*CINAHL (Ebsco platform)*
S79 S76 AND S77
S78 S76 AND S77
S77 S28 OR S49 OR S67
S76 S13 AND S75
S75 S69 OR S70 OR S71 OR S72 OR S73 OR S74
S74 SO (canada or canadian* or “british columbia” or “colombie brittanique” or saskatchewan or manitoba or ontario or quebec or “new brunswick” or “nouveau brunswick” or “nova scotia” or “nouvelle ecosse” or “prince edward island” or “ile du prince edouard” or newfoundland or “terre neuve” or labrador or nunavut or nwt or “territoires du nord ouest” or “northwest territor*” or yukon or “north america” or toronto or montreal or vancouver or ottawa” or edmonton or “quebec city” or winnipeg or hamilton…
S73 (canada or canadian* or “british columbia” or “colombie brittanique” or saskatchewan or manitoba or ontario or quebec or “new brunswick” or “nouveau brunswick” or “nova scotia” or “nouvelle ecosse” or “prince edward island” or “ile du prince edouard” or newfoundland or “terre neuve” or labrador or nunavut or nwt or “territoires du nord ouest” or “northwest territor*” or yukon or “north america*” or toronto or montreal or calgary or ottawa or edmonton or winnipeg or vancouver or gatineau or “queb…
S72 (canada or canadian* or alberta or “british columbia” or “colombie brittanique” or saskatchewan or manitoba or ontario or quebec or “new brunswick” or “nouveau brunswick” or “nova scotia” or “nouvelle ecosse” or “prince edward island” or “ile du prince edouard” or newfoundland or “terre neuve” or labrador or nun?v?t or nwt or “territoires du nord ouest” or yukon)”
S71 (MH “Aboriginal Canadians+”)
S70 (MH “North America+”)
S69 (MH “Canada+”)
S68 S29 AND S49
S67 S64 OR S65 OR S66
S66 (MM “Intersectionality”)
S65 S53 AND S62
S64 S53 AND S62
S63 s53 NOT s59
S62 S60 OR S61
S61 “work outside the home”
S60 “work onsite”
S59 S54 OR S55 OR S56 OR S57 OR S58
S58 “shelter at home”
S57 (MM “Telecommuting”)
S56 “work at home”
S55 “work from home”
S54 “work remotely”
S53 S50 OR S51 OR S52
S52 TX (trustee* or astronaut* or ethicist* or “foreign professional personnel” or “government employee*” or “ metal worker*” or miner* or police or monk* or nun* or “firefighter* or “sex worker*”)
S51 (MM “Case Managers”) OR (MM “Correctional Facilities Personnel”) OR (MM “Doulas”) OR (MM “Farmworkers”) OR (MH “Military Personnel+”) OR (MM “Pilots”) OR (MM “Blue Collar Workers”)
S50 TX “essential worker*”
S49 S29 OR S30 OR S31 OR S32 OR S33 OR S34 OR S35 OR S36 OR S37 OR S38 OR S39 OR S40 OR S41 OR S42 OR S43 OR S44 OR S45 OR S46 OR S47 OR S48
S48 TX (“economic disparit*” or “structural inequality” or “food insecurity” or deficienc* or “lack of social security” or unemploy* or welfare or intersectionality or “health inequity” or “health inequality”)
S47 TX (homeless or “loss or dwelling” or unsheltered or “lack of shelter” or “in need of housing” or “housing deprived”)
S46 “sociology problems”
S45 “working poor”
S44 (MM “Homeless Persons”) OR (MM “Indigent Persons”) OR (MM “Medically Underserved”) OR (MM “Medically Uninsured”) OR (MM “Minority Groups”) OR (MM “Transients and Migrants”)
S43 “ill-housed persons”
S42 “social stigma”
S41 “social marginalization”
S40 (MM “Work-Life Balance”)
S39 (MM “Comfort”)
S38 (MH “Social Class+”) OR (MM “Low Socioeconomic Status”) OR (MM “Social Mobility”)
S37 (MM “Housing Instability”)
S36 “economic stability”
S35 (MH “Socioeconomic Factors+”) OR (MM “Economic Factors”) OR (MM “Economic Status”) OR (MM “Educational Status”) OR (MM “Housing Instability”) OR (MM “Low Socioeconomic Status”) OR (MH “Social Class+”) OR (MM “Social Mobility”) OR (MM “Unemployment”)
S34 TX “child poverty”
S33 (MM “Child Welfare”)
S32 (MM “Poverty”)
S31 TX (“low income” or “low-income” or needy or indigent or impoverished or destitute or penniless or “poverty-stricken” or “poverty stricken” or necessitous or straitened or “lower class” or “lower-class”)
S30 (MM “Public Housing”)
S29 (MH “Europeans+”) OR (MM “Eastern Europeans”) OR (MM “Scandinavians and Nordic Persons”) OR (MM “White Persons”)
S28 S14 OR S15 OR S16 OR S17 OR S18 OR S19 OR S20 OR S21 OR S22 OR S23 OR S24 OR S25 OR S26 OR S27
S27 TX “black communit*” or “health inequity” or “health inequality” or “black Canadian*” or “african canadian” or caribbean or “caribbean and black commmunit* racial disparit*” or intersectionality or race
S26 (MM “Stereotyping”)
S25 (MH “Racism+”) OR (MM “Systemic Racism”)
S24 TX dene
S23 TX cree OR TX haudenosaunee OR TX metis
S22 TX “first nation*”
S21 (MH “Ethnic Groups+”) OR (MH “Africans+”) OR (MM “Arabs”) OR (MH “Asians+”) OR (MH “Australasians+”) OR (MH “Black Persons+”) OR (MH “Indigenous Peoples+”) OR (MH “Jews+”) OR (MM “Kurds”) OR (MM “Middle Eastern Persons”) OR (MH “North Americans+”) OR (MM “Aboriginal Canadians”) OR (MH “African Americans”) OR (MM “Amish”) OR (MH “Hispanic Americans”) OR (MM “Inuit”) OR (MH “Native Americans”) OR (MM “Roma”) OR (MM “South Americans”)
S20 (MM “Special Populations”)
S19 (MM “Minority Groups”)
S18 “health disparate”
S17 (MM “Refugees”)
S16 “population groups”
S15 (MH “Undocumented Immigrants”)
S14 (MM “Emigration and Immigration”)
S13 S1 OR S2 OR S3 OR S4 OR S5 OR S6 OR S7 OR S8 OR S9 OR S11 OR S12
S12 TX “sars cov 2”
S11 TX “sever acute respiratory syndrome coronavirus 2” OR TX “severe acute respiratory syndrome coronavirus 2” OR TX wuhan
S10 TX “2019-nCov disease” OR TX “2019 ncov infection” OR TX “coronavirus disease 19”
S9   TX 2019-nCoV OR TX COVID-19 OR TX “2019 novel coronavirus disease”
S8   TX CoV OR TX SARS-CoV OR TX MERS-CoV
S7   TX “middle east respiratory syndrome” OR TX severe acute respiratory syndrome” AND TX SARS
S6   TX coronavirus* OR TX “corona virus” OR TX mers
S5   MH covid 19
S4   MW covid
S3   (MH “SARS Virus”)
S2  (MH “Coronavirus Infections+”) OR (MM “Middle East Respiratory Syndrome”) OR (MH “COVID-19+”) OR (MH “Coronavirus+”) OR (MH “SARS-CoV-2”)
S1   MH coronavirus

## Appendix B. Grey Literature Search Terms

**Context (Applied in Conjugation with the Boolean Operator “AND”)**	**Target Location (Applied in Conjugation with the Boolean Operator “AND”)**	**Target Interest (Applied in Conjugation with the Boolean Operator “AND”)**	**Target Population (Applied in Conjugation with the Boolean Operator “AND”)**
COVID-19	Canada	impact	racialized communities
COVID-19	Canada	impact	homeless
COVID-19	Canada	impact	essential workers (Boolean operator NOT healthcare workers)
COVID-19	Canada	impact	African communities
COVID-19	Canada	impact	migrant workers
COVID-19	Canada	impact	low-income
COVID-19	Canada	impact	truckers
COVID-19	Canada	impact	Caribbean communities
COVID-19	Canada	impact	racial + minoritized
COVID-19	Canada	impact	housing challenged
COVID-19	Canada	impact	Asian communities
COVID-19	Canada	impact	South Asian communities
COVID-19	Canada	impact	South American
COVID-19	Canada	impact	North African communities
COVID-19	Canada	impact	Middle eastern
COVID-19	Canada	impact	grocery clerks
COVID-19	Canada	impact	minimum wage workers
COVID-19	Canada	impact	displaced refugees
COVID-19	Canada	impact	transient shelter
COVID-19	Canada	impact	retail workers
COVID-19	Canada	impact	restaurant staff
COVID-19	Canada	impact	farmers
COVID-19	Canada	impact	immigrants
COVID-19	Canada	impact	Indigenous

## Appendix C. Data Extraction Instructions and Data Fields

There are two sections to the data extraction form.

Section 1—general information about the citation/reference.

Section 2—details about the citation/reference.

General information	
Title	
Lead author	
Year of publication	
Publication source	
Countries in which the study conducted	
Province/territory in which the study conducted	
Study Characteristics	
Methods	Aim of study
	Study design - Quantitative - Qualitative - Mix-method - Systematic review - Other
	Details of study design
	Data source
	Context
Participants	Total population size (N)
	Population description
	Sample size of interest (n)
	Age
	Gender
	Income
	Race/Ethnicity
	Other demographic information
Outcomes	Reported Impact on Health outcomes (quantitative)
	Reported Impact on Health outcomes (qualitative)
	Reported Impact on Social Determinants of Health (SDoH) (quantitative)
	Reported Impact on Social Determinants of Health (SDoH) (qualitative)
	Reported Impact on Health inequities (quantitative)
	Reported Impact on Health inequities (qualitative)
	Comparator(s) (group/outcomes)
	Reported Effect on Comparator
	Other sociodemographic variables
